# Spatio-Temporal Dynamics of Lettuce Metabolome: A Framework for Targeted Nutritional Quality Improvement

**DOI:** 10.3390/plants13233316

**Published:** 2024-11-26

**Authors:** Ivan Simko

**Affiliations:** Sam Farr United States Crop Improvement and Protection Research Center, Agricultural Research Service, U.S. Department of Agriculture, Salinas, CA 93905, USA; ivan.simko@usda.gov

**Keywords:** *Lactuca*, metabolites, spatial dynamics, temporal dynamics, leaf development, antioxidants, nutritional quality, head closure

## Abstract

Lettuce (*Lactuca sativa* L.) is a popular leafy vegetable valued for its dietary fiber, antioxidants, and beneficial vitamins. This study presents a comprehensive spatio-temporal analysis of the lettuce metabolome, revealing complex dynamics in metabolite accumulation influenced by plant age, leaf position, proximodistal distribution within a leaf, and head closure. Samples were collected from plants at five maturity stages (ranging from baby leaf to full commercial maturity and eventually to bolting) and from five leaf positions (from the apex to the base of each plant). A widely targeted metabolomics approach identified 1905 compounds, with flavonoids, phenolic acids, and lipids as the largest classes. Younger plants exhibited higher levels of flavonoids, while older plants accumulated more saccharides and amino acids. Metabolites showed distinct proximodistal distributions, with flavonoids and vitamins concentrated at leaf tips and terpenoids declining from base to tip. Head closure significantly reduced levels of flavonoids, retinol (vitamin A1), and riboflavin (vitamin B2), while it was associated with increased content of other beneficial vitamins, such as thiamine (B1), pantothenate (B5), and pyridoxine (B6). Broad-sense heritability (*H*^2^) estimates for metabolites yielded mean *H*^2^ values of 0.648 and 0.743 for plants at baby-leaf and commercial maturity stages, respectively. The overall highest heritability was observed in tannins (*H*^2^ = 0.909) in younger plants and chalcones (*H*^2^ = 0.894) in older plants, suggesting strong genetic control over specific metabolite classes and subclasses. These findings offer a robust framework for optimizing lettuce’s nutritional profile by linking metabolite distributions to developmental processes, plant architecture, and genetic regulation. By leveraging these insights, breeders and producers can develop targeted strategies to enhance metabolite content through optimized breeding and harvesting strategies.

## 1. Introduction

Lettuce (*Lactuca sativa* L.) is one of the most economically important leafy vegetables worldwide, valued for its nutritional content, including vitamins, dietary fiber, flavonoids, and various bioactive compounds [1,2]. Regular consumption of fruits and vegetables, including lettuce, is associated with a reduced risk of chronic metabolic diseases such as obesity, cardiovascular disease, and diabetes [3,4]. Flavonoids, in particular, have been shown to provide significant health benefits. For example, quercetin and its monoglucosides have been linked to a reduction in deaths due to cardiovascular diseases and have been shown to inhibit 15-lipoxygenase, an enzyme involved in the development of atherosclerosis [5,6]. The nutritional benefits of lettuce are further enhanced by its content of dietary minerals, vitamins, and carotenoids, all of which contribute to its value as a staple in healthy diets [1,7].

While traditional lettuce breeding programs have primarily focused on developing cultivars that are resistant to pests, pathogens, and physiological disorders, as well as those with high yield and long shelf life, recent efforts have aimed to enhance the phytochemical content of lettuce, particularly its phenolic compounds and flavonoids [8]. Lettuce exhibits substantial variation in the levels of these phytochemicals among different cultivars, including derivatives of caffeic acid, such as chlorogenic, chicoric, caffeoyltartaric, and caffeoylmalic acids, as well as flavonol glycosides like quercetin 3-O-malonylglucoside, quercetin 3-O-glucoside, and quercetin 3-O-glucuronide [9,10]. The genetic potential for breeding lettuce cultivars with elevated levels of these bioactive compounds is present in the existing germplasm [11], providing opportunities for the development of novel, nutritionally enhanced cultivars.

Despite the advances in understanding the genetic architecture of primary metabolism [12], the variation in metabolite profiles across different gene pools [13], and the identification of metabolites associated with postharvest browning [14], comprehensive spatio-temporal analyses of metabolite accumulation patterns in lettuce are still lacking. Understanding how these patterns are influenced by factors such as plant age, leaf position, proximodistal distribution within a leaf, and head closure is crucial for optimizing the nutritional quality and health benefits of lettuce. Lettuce is an ideal model for investigating spatio-temporal patterns of metabolite accumulation due to its distinct growth characteristics and plant architecture, such as dense, overlapping leaves and head formation. These features allow for unique variations in metabolite concentrations across different plant parts and stages of development. Understanding how metabolites are distributed spatially within plants and individual leaves and temporally as the plant grows could offer critical insights into optimizing nutritional content. This information can help identify the specific parts of the plant or developmental stages where key bioactive compounds, like flavonoids, vitamins, and phenolic acids, are most concentrated, thereby guiding breeding strategies and harvesting practices aimed at maximizing nutritional benefits.

Moreover, the practical applications of such spatio-temporal data are significant. For instance, by pinpointing the optimal harvest time and plant regions richest in health-promoting compounds, lettuce cultivars can be developed or managed to better meet dietary needs, thus enhancing its role in promoting human health. This study addresses this gap by providing a detailed analysis of metabolite accumulation in lettuce, offering new insights into the complex interactions between plant development and metabolite production.

## 2. Materials and Methods

### 2.1. Plant Field Cultivation

Field experiments were conducted in the Salinas Valley of California, a primary U.S. commercial lettuce production area. Lettuce plants were grown on raised beds (approximately 1 m wide and 25 cm high) under standard agronomical practices for the area. Seed lines were spaced about 28 cm apart, with plants grown at a final spacing of approximately 30 cm within each line. Before planting, a 6N-20P-20K fertilizer was applied at a rate of 336 kg·ha⁻^1^. After planting, an anticrustant fertilizer (7N–7P–0K–7.46S) was sprayed to prevent crust formation on the soil surface. Ammonium sulfate (21N–0P–0K–24S) was then applied at 336 kg·ha⁻^1^ at both 28 and 42 days after planting (DAP). Irrigation was provided using overhead sprinklers three times per week until seedling thinning at 25 DAP, after which watering was reduced to twice weekly for the remainder of the experiment. Two parallel experiments described in Section 2.2 and Section 2.3 were conducted.

### 2.2. Experiment 1: Spatio-Temporal Dynamics

**Figure 1:**
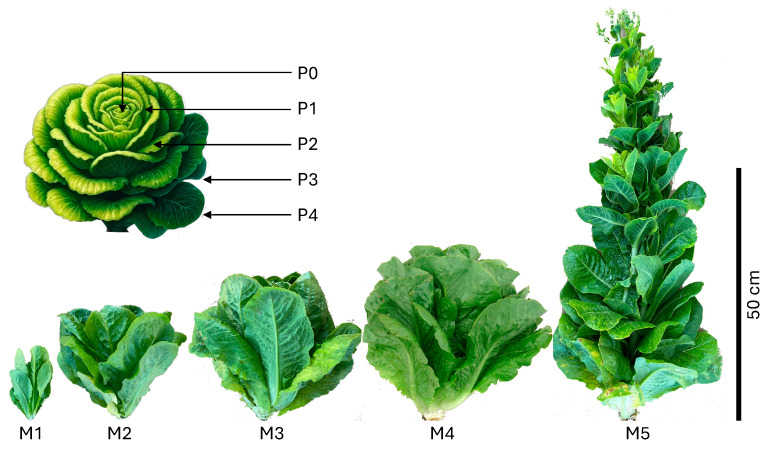
Growth, development, and sampling of lettuce SM09B plants for spatio-temporal analysis. Plants were harvested at five maturity stages (M1 to M5) over a 48-day period, from 30 to 78 days after planting (DAP). Five individual leaves were collected from different positions (P0–P4) on each plant, as shown in the illustration. To improve clarity, the background was removed before combining photographs of the plants from different maturity stages. The leaf sampling positions are indicated in the illustration, which was created using Microsoft Copilot (Microsoft Corporation, powered by DALL·E, OpenAI, San Francisco, CA, USA).

Romaine lettuce plants from breeding line SM09B [15] were harvested at five timepoints (maturity M1 to M5) over 48 days, from 30 to 78 DAP, at 12-day intervals. M1 represented the commercial baby-leaf stage, M4 corresponded to full market maturity, and M5 was considered overmature, marked by plant bolting. Although M2 and M3 stages are not typically harvested commercially, they were included in this study to capture the metabolome transition from M1 to M4. The early stages of head closing in the SM09B line begin at the M3 stage (or shortly thereafter) and continue into M4, when the heads are fully closed (Figure 1). At each stage, three representative plants of similar size were harvested. Measurements included plant height, fresh weight, and number of leaves longer than 7 cm.

Five individual leaves were collected from different positions on the plant (P0–P4): P0 leaves were near the apex and shorter than 7 cm; P1 leaves were the youngest, longer than 7 cm, and located in the top 25% of the plant; P2 leaves were from the upper middle 25–50% of the plant; P3 leaves were from the lower middle 50–75% of the plant; and P4 leaves were the oldest, located in the bottom 25% of the plant, all longer than 7 cm (Figure 1). Additionally, at the market maturity (M4) stage, P2 leaves from randomly selected eight plants were divided into tip and base sections to analyze metabolite distribution along the proximodistal axis.

### 2.3. Experiment 2: Accession Diversity and Metabolites Heritability

Forty-five lettuce accessions were grown under identical conditions as in Experiment 1 and harvested at baby-leaf (M1, 30 DAP) and market maturity (M4, 66 DAP) stages. P2 leaves were collected to analyze metabolite concentration differences between maturity stages and determine compound level correlations. A subset of 20 accessions, with three evaluated plants per accession, was used to calculate the broad-sense heritability (*H*^2^) of each metabolite.

### 2.4. Sample Processing and Metabolomic Analysis

Immediately after harvest, plant samples were flash-frozen in liquid nitrogen, stored at −80 °C, and shipped to Metware Biotechnology (Woburn, MA, USA) for widely targeted metabolomic analysis [16]. The samples were lyophilized using a Scientz-100 F lyophilizer, ground with zirconia beads (MM 400 Retsch, 30 Hz, 1.5 min, Retsch GmbH, Haan, Germany), and dissolved in 1200 µL of 70% methanol pre-cooled to −20 °C, containing internal standards. The mixture was vortexed six times for 30 s every 30 min, then centrifuged at 12,000 RPM for 3 min at 4 °C). The supernatant was incubated overnight at 4 °C, collected, and filtered through a 0.22 µm filter membrane before being stored for ultra-performance liquid chromatography (UPLC) tandem mass spectrometry (MS/MS) analysis.

UPLC was performed using an ExionLC™AD system (SCIEX, Framingham, MA, USA) with an Agilent SB-C18 column (1.8 µm, 2.1 × 100 mm, Agilent Technologies, Santa Clara, CA, USA) at 40 °C. The mobile phases consisted of ultrapure water (A) and acetonitrile (B), both containing 0.1% formic acid. The gradient elution was programmed to increase from 5% to 95% B over 9 min, with a total run time of 14 min. The flow rate was set at 0.35 mL/min at 40 °C.

Mass spectrometry was conducted using a QTRAP 6500 system (SCIEX), operating in both linear ion trap (LIT) and triple quadrupole (QQQ) modes. This system is equipped with an ESI Turbo ion spray interface and both positive and negative ion modes were controlled by Analyst 1.6.3 software (SCIEX). The following ion source conditions were applied: source temperature 550 °C; ion spray voltage (IS) 5500 V (positive ion mode)/−4500 V (negative ion mode); and gas pressures (GSI, GSII, CUR) set to 50, 60, and 25 psi, respectively. Metabolites were quantified in multiple reaction monitoring (MRM) mode.

Metabolite identification was performed using an in-house database from the Metware Biotechnology, supplemented with secondary mass spectrometry data. Identification was based on the precise mass of metabolites, MS2 fragments, isotopic distribution, and retention time (RT) of MS2 fragments. Quantification was carried out by triple quadrupole scanning (QQQ) based on comprehensive targeted metabolomics techniques.

Instrument stability was verified by analyzing quality control (QC) samples. QC samples were prepared by pooling equal aliquots from 10 randomly selected real samples, with each real sample used only once in the QC. The initial data were processed using MultiQuant 3.0.3 software (SCIEX), followed by further analysis using XCMS software 4.4.0 within the R environment (https://www.R-project.org/, accessed on 26 January 2024) for peak alignment, retention time correction, and peak area extraction. Differentially abundant metabolites were identified using orthogonal partial least squares discriminant analysis (OPLS-DA) with 200 permutations for model validation. The following significance criteria were applied: variable importance in projection (VIP) ≥ 1, fold change ≥ 2 or ≤0.5, and *p*-value < 0.05.

Identified metabolites were annotated using the Kyoto Encyclopedia of Genes and Genomes (KEGG) database (http://www.kegg.jp/kegg/compound/, accessed on 26 January 2024) [17]. These metabolites were then mapped to the KEGG Pathway database (http://www.kegg.jp/kegg/pathway.html, accessed on 26 January 2024). KEGG pathway enrichment was calculated using the hypergeometric test.

### 2.5. Statistical Analyses and Graphs Plotting

Due to the skewed distribution of most metabolites, data were transformed using inverse normal transformation (INT) before statistical analyses [18]. INT transforms the sample distribution to approximate a normal distribution with a mean of 0 and a standard deviation of 1. This transformation normalizes the distribution of metabolite concentrations, facilitating comparative analysis across different compounds and samples.

Analyses included hierarchical clustering of metabolites using spatio-temporal data from Experiment 1, paired *t*-tests to assess differences in metabolite content between young and mature plants (Experiment 2) and leaf proximodistal distribution (tip vs. base; Experiment 1), Pearson correlations to examine relationships between metabolite content in young and old plants, as well as between the leaf tip and base, and a Chi-Square ‘Goodness of Fit’ test to assess the distribution of metabolite classes and subclasses into clusters. All analyses were adjusted for multiple comparisons using the false discovery rate (FDR) approach and performed using JMP Pro 17 (SAS Institute, Cary, NC, USA). For metabolite classes and subclasses, statistical significance was determined using 10,000 bootstrap replicates.

Broad-sense heritability (*H*^2^) was calculated for each metabolite using data from the subset of 20 accessions in Experiment 2. While these estimates provide insights into the genetic control of metabolite production under study conditions, they may not fully represent the range of potential genetic and environmental interactions.

For spatio-temporal graphs, smoothing lines were generated using 1000 random forest analyses for each graph, with predicted data used for plotting.

## 3. Results and Discussion

### 3.1. Metabolite Profiles and Accumulation Patterns

Two field experiments were conducted. In the first experiment, romaine lettuce plants used for spatio-temporal analysis were harvested at five timepoints 12 days apart (Figure 1). The M1 stage (30 DAP) represented the baby-leaf phase, M2 (42 DAP) and M3 (54 DAP) are transitional stages not typically harvested commercially, M4 (66 DAP) corresponded to market maturity, and M5 (78 DAP) was considered overmature, marked by plant bolting. The second experiment examined 45 lettuce accessions at two maturity stages (M1 and M4) to assess genetic diversity and heritability.

**Figure 2:**
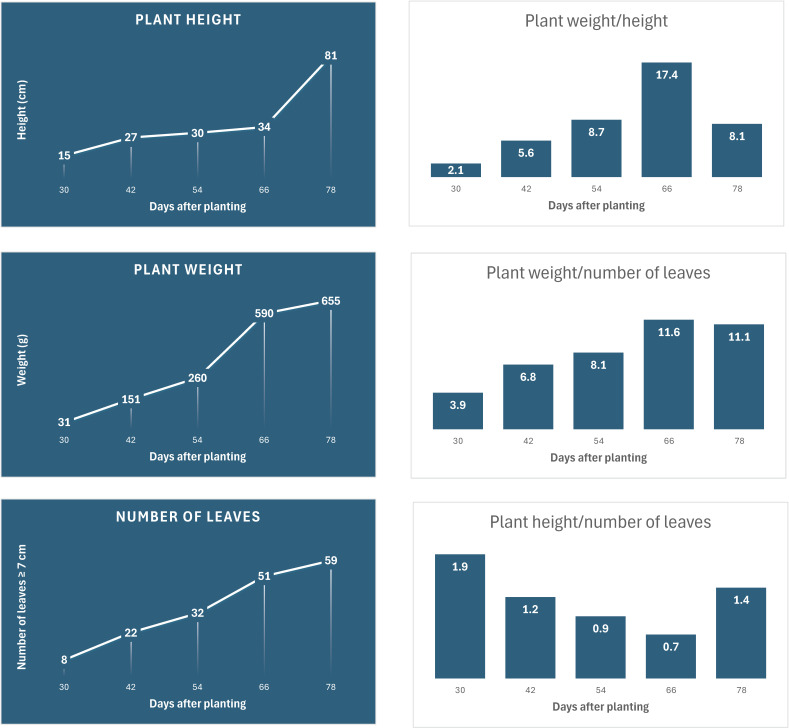
Size of SM09B line plants used for widely targeted metabolomic analysis harvested at five timepoints.

At the M1 stage (30 DAP), SM09B plants weighed on average about 31 g, were 15 cm tall, and produced 8 leaves longer than 7 cm (Figure 2). A gradual increase in plant height, weight, and the number of leaves (>7 cm) was observed between the M1 and M3 stages. A more substantial increase in fresh weight occurred between the M3 (54 DAP) and M4 (66 DAP) stages, when plants experienced rapid biomass accumulation (from 260 g to 590 g), an increase in leaf number (from 32 to 51), and the formation of closed heads. After reaching market maturity (M4), plant height increased substantially (from 34 cm to 81 cm) as plants transitioned from the vegetative to the reproductive stage and began bolting (M5) (Figure 1 and Figure 2). The transition from M4 to M5 was accompanied by a modest increase in biomass (from 590 g to 655 g), with small leaves emerging on the elongated stem. The ratio of plant weight to height and to leaf number gradually increased from M1 to M4 (from 2.1 to 17.4, and from 3.9 to 11.6, respectively), while the ratio of height to leaf number decreased (from 1.9 to 0.7), indicating that the plants were becoming more compact and the average leaf weight increased with age. This trend reversed as plants began to bolt, forming new small leaves on the elongated stem (Figure 2).

**Figure 3:**
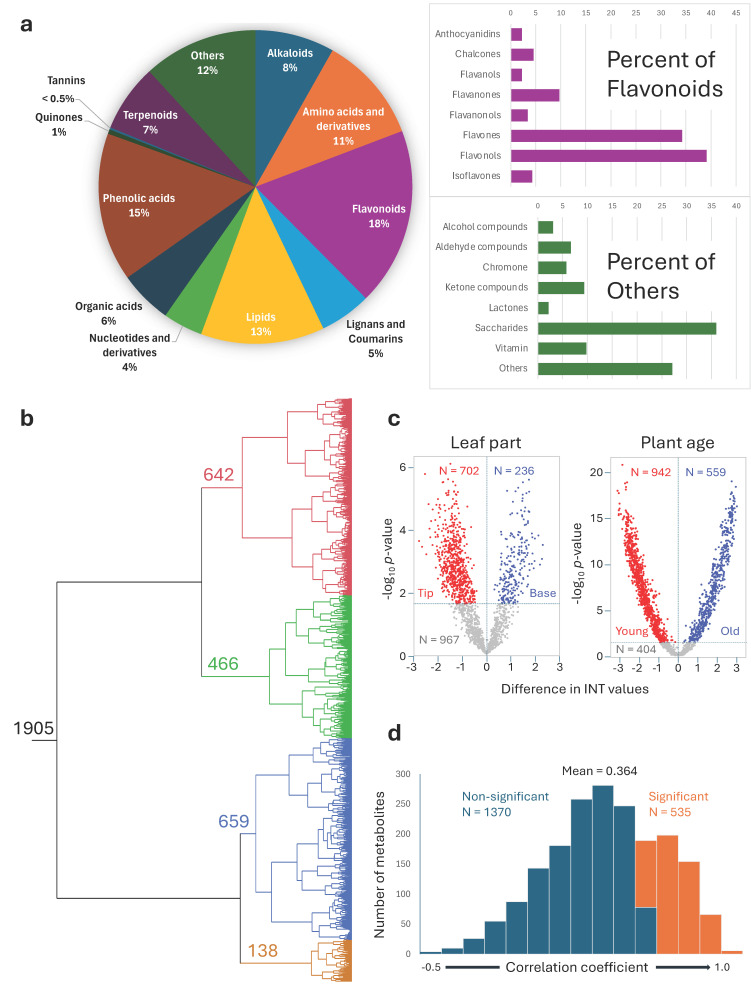
Dynamics of the lettuce metabolome. (**a**) Relative abundance of metabolite classes (circular graph) and subclasses (bar graphs) among 1905 compounds detected in lettuce. (**b**) Hierarchical clustering of compounds into four major clusters based on their spatio-temporal dynamics in SM09B. (**c**) Relative abundance of metabolites in leaf parts (tip or base half of a leaf) of SM09B and plants of different ages (young—baby-leaf stage—30 DAP, and old—market maturity—66 DAP) of 45 accessions. Metabolites with significantly (*p* < 0.05) higher content in leaf tips or younger plants are in red, and those significantly higher in the leaf base or older plants are in blue. INT values represent the inverse normal transformed metabolite abundances. (**d**) Distribution of correlation coefficients between metabolites detected in younger and older plants of 45 accessions.

A comprehensive metabolomic analysis of plants from both experiments identified 1905 metabolites (Table S1), with flavonoids (18%), phenolic acids (15%), and lipids (13%) as the largest classes. Within the flavonoid class, flavonols and flavones were the most abundant subclasses (Figure 3a), consistent with previous findings [19]. Hierarchical clustering revealed four major metabolite clusters based on distinct accumulation patterns across SM09B tissues and developmental stages (Figure 3b, Table 1). These four spacio-temporal clusters accounted for approximately 33.7%, 24.5%, 34.6%, and 7.2% of all metabolites, respectively. Metabolites within each cluster are presumed to exhibit comparable spatio-temporal accumulation patterns. Notable deviations from the expected distributions were observed in several classes and subclasses of metabolites. In cluster I, isoflavones and ketone compounds were notably overrepresented. In cluster II, overrepresentation was observed for anthocyanidins, lactones, chalcones, and flavanones. In cluster III, amino acids and derivatives, lipids, organic acids, nucleotides and derivatives, and tannins were overrepresented. Finally, cluster IV showed an overrepresentation of terpenoids (Table 1). However, not all differences in distribution to the four clusters were statistically significant from the overall mean, due to the small number of metabolites in certain classes and subclasses. Chi-square tests yielded the most highly significant results for terpenoids (*p* = 1.2 × 10^−41^), amino acids and derivatives (*p* = 1.6 × 10^−35^), flavonoids (*p* = 3.1 × 10^−33^), lipids (*p* = 4.8 × 10^−15^), flavonols (*p* = 1.8 × 10^−12^), phenolic acids (*p* = 2.6 × 10^−12^), and flavones (*p* = 1.1 × 10^−11^).

### 3.2. Spatio-Temporal Distribution of Metabolites

Describing the distribution of nearly 2000 metabolites detected in lettuce (Table S1) in detail is not feasible; therefore, the focus is on the spatio-temporal distribution of selected metabolites, their subclasses, and classes that are vital for human health (e.g., flavonoids and vitamins), lettuce taste (e.g., terpenoids and saccharides), and plant growth and development (e.g., phytohormones).

**Figure 4:**
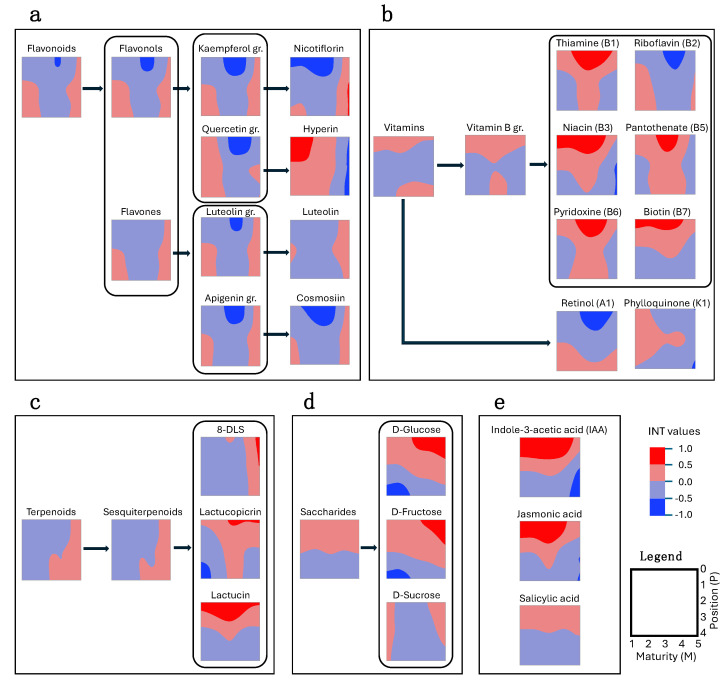
Spatio-temporal distribution of lettuce metabolites. This figure illustrates the distribution patterns of various metabolites in lettuce plants across different growth stages and leaf positions. Examples are provided for selected classes, subclasses, groups, and individual compounds. X-axis indicates plant maturity ranging from baby-leaf stage (M1, 30 DAP) to bolting stage (M5, 78 DAP), progressing from left to right. Y-axis indicates the leaf position on the plant from youngest (P0, top) to oldest (P4, bottom) leaves. INT values represent the inverse normal transformed metabolite abundances. Metabolite abundance is represented by a color gradient: dark red indicates the highest abundance, while dark blue indicates the lowest abundance. Intermediate colors represent varying levels of abundance between these extremes. (**a**) flavonoids; (**b**) vitamins; (**c**) terpenoids; (**d**) saccharides; (**e**) phytohormones.

The analysis of metabolite dynamics, considering both plant age and leaf position, revealed that head closure significantly affected metabolite content, with many flavonoids exhibiting reduced concentrations in closed heads (Figure 4a). This was likely due to limited leaf exposure to ultraviolet radiation, which plays a crucial role in the accumulation of flavonoids, anthocyanins, and methoxycinnamic acid [20]. Vitamins displayed diverse accumulation patterns across plant development and leaf position. Thiamine (B1), niacin (B3), pantothenate (B5), pyridoxine (B6), and biotin (B7) were highest in younger leaves and closed heads (Figure 4b), while riboflavin (B2) and retinol (A1) declined in closed heads (Figure 4b). This aligns with previous findings of decreased β-carotene and vitamin C in artificially closed romaine lettuce heads [21]. Terpenoids, especially those associated with bitterness like 8-deoxylactuin-15-sulphate (8-DLS), increased with plant age and accumulated as plants approached bolting (Figure 4c), corresponding with previous studies linking these compounds to bitterness in lettuce [22,23,24]. Saccharides were generally more abundant in younger leaves, but glucose and fructose accumulated predominantly in older plants transitioning to the reproductive stage, particularly in their younger tissue (Figure 4d). This pattern may reflect changes in source–sink relationships and carbon allocation as the plant matures. Phytohormones such as indole-3-acetic acid (IAA), jasmonic acid, and salicylic acid primarily accumulated in young leaves of younger plants (Figure 4e), aligning with their critical role in regulating plant growth and development [25,26,27].

### 3.3. Proximodistal Distribution of Metabolites

The distribution of metabolites along the proximodistal axis of leaves from the upper-middle (P2) region of SM09B line plants at marker maturity (M4) was analyzed in eight leaves. A total of 702 metabolites were significantly (*p* < 0.05) more abundant in the leaf tips, while 236 were more abundant in the leaf bases (Figure 3c). Flavonoids (particularly flavonols) and vitamins were concentrated in the tips, while terpenoids showed a decline from base to tip (Table 1). This proximodistal distribution likely reflects differential exposure to light [28], as well as other environmental [29,30] and intrinsic factors [31], across the leaf surface, consistent with findings in differently pigmented lettuce leaves [19].

**Figure 5:**
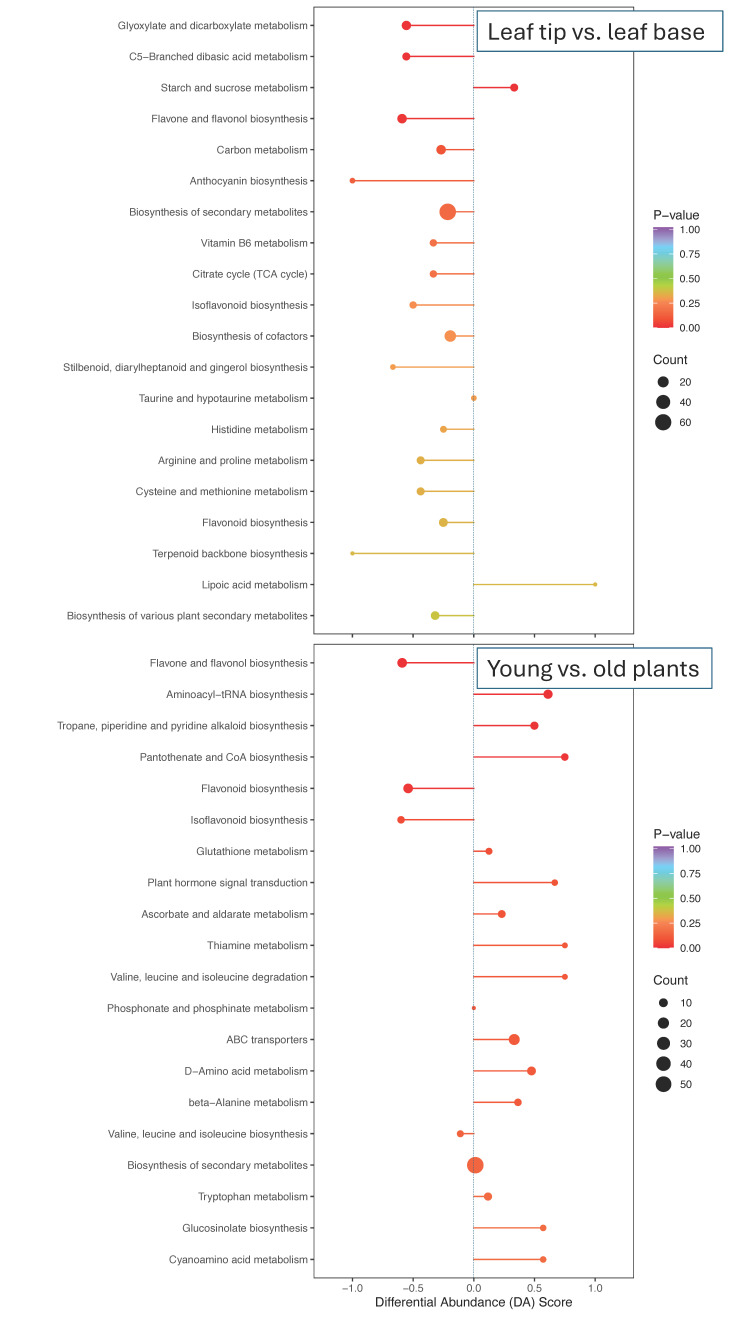
Overall changes in KEGG metabolic pathway. The top panel shows results comparing leaf tip vs. base, while the bottom panel shows results comparing young (30 DAP) vs. old (66 DAP) plants. The Y-axis shows the name of the differential pathway, and the X-axis represents the differential abundance (DA) score. DA score, which is calculated as the difference between the number of upregulated and downregulated metabolites in a pathway, divided by the total number of metabolites annotated in the pathway, reflects the overall change of all metabolites in the metabolic pathway. A positive DA score indicates that the expression trend of all identified metabolites in this pathway is up-regulated in leaf base and older plants, respectively, while the negative score indicates that the expression trend of all identified metabolites in this pathway is up-regulated in leaf tips and younger plants, respectively. The length of the line represents the absolute value of DA score while the size of the dot at the end of the line represents the number of differential metabolites. The color of the line and the dot represent the *p*-value.

Among the top 20 metabolic pathways showing differences between the leaf tip and base, only two exhibited upregulation in the leaf base, starch and sucrose metabolism and lipoic acid metabolism, while all other pathways were upregulated in the leaf tips (Figure 5). Notable pathways upregulated in the leaf tip important for the production of compounds beneficial to human health include flavone and flavonol biosynthesis, anthocyanin biosynthesis, vitamin B6 metabolism, isoflavonoid biosynthesis, and general flavonoid biosynthesis. The higher accumulation of saccharides in the leaf tips (Table 1), despite the upregulation of the starch and sucrose metabolism pathway in the leaf base (Figure 5), is somewhat unexpected. However, since the basal parts of lettuce P2 leaves at the M4 maturity stage are substantially shaded, this discrepancy may reflect a typical source–sink relationship [32], where the leaf tip produces sugars via photosynthesis, while the shaded base is involved in sucrose metabolism—either storing, converting, or transporting sugars.

The average correlation in metabolite abundance between the leaf tip and base was 0.325. The highest correlations were found for the following classes and subclasses of metabolites: saccharides (r = 0.590), chromones (r = 0.519), terpenoids (r = 0.454), and isoflavones (r = 0.433) (Table 1). High correlations in certain metabolite classes between the two leaf regions suggest relatively stable abundances across the leaf, even though the absolute levels of metabolites may differ. This stability can be advantageous for plant breeders as it indicates a more consistent metabolic trait across the leaf, simplifying selection for desired characteristics.

The proximodistal distribution of metabolites in lettuce leaves, along with the results from the analysis of metabolic pathways, suggests a leaf region-specific specialization of metabolic processes, likely influenced by both environmental and intrinsic factors. The leaf tips are particularly enriched with compounds that are important for both plant function and human nutrition. These findings may have implications for agricultural practices aimed at optimizing the nutritional content of lettuce, such as ensuring that plants receive sufficient light during growth.

### 3.4. Plant Age-Related Differences

Plants from 45 lettuce accessions were harvested at baby-leaf (M1, 30 DAP) and market maturity (M4, 66 DAP) stages. P2 leaves were collected to analyze metabolites at different maturity stages and to calculate their broad-sense heritability (*H*^2^).

Plant age-related differences were significant, with 942 metabolites being more abundant in younger plants and 559 in older plants (Figure 3c). Younger plants showed the highest increase in flavonoids, particularly flavanols, isoflavones, and flavanones (Table 1), known for their antioxidant and anti-inflammatory properties [5,10]. Older plants accumulated significantly (*p* < 0.05) more saccharides and amino acids (Table 1), potentially reflecting changes in primary metabolism as the plant matures.

Analysis of the Kyoto Encyclopedia of Genes and Genomes (KEGG) revealed that younger plants exhibit a large proportion of upregulated metabolites in the flavone, flavonol, flavonoid, and isoflavonoid biosynthesis pathways (Figure 5). In contrast, the most upregulated pathways in plants at commercial maturity include plant hormone signal transduction, ascorbate and thiamine metabolism, and valine, leucine, and isoleucine degradation (Figure 5). The upregulation of these pathways at commercial maturity likely reflects the plant’s preparation for the reproductive stage, where energy, nutrient allocation, and hormonal control need to be carefully balanced [33].

The correlation in metabolite abundance between leaves from young and mature plants ranged from −0.475 to 0.950 (mean: 0.364), with 535 metabolites showing a highly significant positive correlation (Figure 3d). Among the metabolite classes and subclasses, the highest correlation was observed for anthocyanins (r = 0.503), followed by tannins (r = 0.476), flavanonols (r = 0.432), and flavonols (r = 0.421) (Table 1). A high correlation between the two maturity stages indicates that, although the absolute levels of metabolites in certain classes and subclasses may differ, their relative abundances are maintained at similar ratios across the tested accessions throughout most or all of the plant production cycle.

Broad-sense heritability (*H*^2^) estimates for individual metabolites ranged from 0 to 0.987 (mean of 0.648) in younger plants and 0 to 0.984 (mean of 0.743) in older plants. For metabolite classes or subclasses, the average *H*^2^ ranged from 0.485 (quinones) to 0.909 (tannins) in young plants, and 0.498 (alcohol compounds) to 0.894 (chalcones) in older plants, suggesting strong genetic control for certain metabolite classes (Table 1). These findings highlight the potential for breeding programs to enhance specific metabolic traits.

### 3.5. A Framework for Targeted Nutritional Quality Improvement

Breeding leafy vegetables for nutritional quality poses unique challenges since all edible parts are harvested and consumed. Unlike crops where only specific parts (e.g., fruits, seeds, or tubers) are consumed, lettuce shows significant variability in metabolite profiles influenced by plant and leaf age, position, and shading from dense architecture. This variability makes it difficult to adopt a single breeding strategy that addresses the diversity of lettuce types, harvest stages, and market demands. The spatio-temporal metabolomics approach in this study reveals a complex interplay between developmental processes, plant architecture, and genetic regulation. Data on 1905 metabolites (Table S1) provide valuable guidance for optimizing specific nutritional or flavor compounds, such as vitamins, flavonoids, sesquiterpenoids, and saccharides, and for identifying ideal harvest times to maximize desired metabolite content.

Table S1 serves as a practical roadmap to guide targeted breeding and production decisions:

1. Determining optimal harvest time. Table S1 highlights how metabolite levels change across maturity stages, from M1 (baby leaf stage) to M5 (bolting). For example, flavonoids like flavonols and chalcones peak during early stages (M1 or M2), while saccharides and certain vitamins are more abundant at commercial maturity (M4). Vitamin B1 (thiamine) levels, for instance, are over four times higher at SM09B market maturity compared to the baby leaf stage, a trend confirmed across 45 accessions. Conversely, vitamins like dehydroascorbic acid are more abundant in younger leaves. Such data allow growers to optimize harvest timing for maximum nutritional content and breeders to develop cultivars suited to specific production systems or market demands.
2. Evaluating genetic diversity and metabolite patterns. Table S1 also incorporates metabolite profiles from 45 accessions at both the M1 and M4 stages. This allows breeders to compare metabolic trends across diverse genetic backgrounds, identifying accessions that exhibit superior levels of specific metabolites at key developmental stages. Such comparisons can guide the selection of parental lines for breeding programs aimed at enhancing nutritional quality.
3. Assessing the impact of head closure: The formation of tightly closed heads during the M4 stage profoundly affects metabolite content due to reduced light exposure. Light-sensitive compounds like flavonoids, riboflavin (B2), and retinol (A1) decrease, while others, such as thiamine (B1) and pyridoxine (B6), increase. Table S1 can help breeders evaluate the effects of head closure on specific metabolites and adjust breeding strategies, such as selecting less compact heads to retain higher levels of light-sensitive compounds.
4. Developing breeding priorities: The spatial variation in metabolite distribution, such as the proximodistal gradients of flavonoids, terpenoids, and saccharides, is another critical resource in Table S1. Breeders can use this information to prioritize traits that align with nutritional or market demands, focusing on genotypes with enhanced metabolite levels in edible portions of the plant.

Despite variability introduced by environmental factors [34] and genetic diversity, this study provides a robust framework for understanding and improving the nutritional profile of lettuce. Future work will involve metabolome profiling 500 lettuce accessions, genotyped with molecular markers [35] and phenotyped for over 25 developmental, physiological, quality, and resistance traits ([36] and several other publications). The goal is to identify accessions with elevated levels of beneficial metabolites for use in production and breeding programs. Metabolome-wide genome-wide association studies (mGWAS) [37] will identify genetic loci associated with beneficial metabolites, elucidating the genetic regulation of key metabolic pathways and supporting predictive breeding models. Together, these efforts will advance strategies to enhance the health benefits, flavor, and market value of lettuce and related leafy vegetables.

## Figures and Tables

**Table 1:** Classes and subclasses of 1905 lettuce metabolites and their distribution in leaf tip and base and in baby-leaf and commercial maturity stage plants.

Class or Subclass of Metabolites	N	Spatio-Temporal Cluster ^1^				Tip vs. Base of Leaves ^2^		Young vs. Old Plants ^2^		Heritability (*H*^2^) ^3^	
		I	II	II	IV	Diff.	Correl.	Diff.	Correl.	Young	Old
Alkaloids	156	49	29	73	5	0.35 ^H^	0.370 ^H^	−0.01	0.367 ^H^	0.591	0.693
Amino acids and derivatives	210	24	22	161	3	0.41 ^H^	0.384 ^H^	−0.61 ^L^	0.369 ^H^	0.627	0.699
Flavonoids	350	188	130	24	8	0.83 ^H^	0.206 ^H^	1.46 ^H^	0.394 ^H^	0.626	0.831
-Anthocyanidins	8	3	5	0	0	0.88 ^H^	0.065	1.22 ^H^	0.503 ^H^	0.739	0.759
-Chalcones	16	7	9	0	0	0.71 ^H^	0.057	1.68 ^H^	0.339 ^H^	0.810	0.894
-Flavanols	8	4	3	0	1	0.82 ^H^	−0.060	1.86 ^H^	0.395 ^H^	0.512	0.747
-Flavanones	34	15	18	1	0	0.75 ^H^	0.150	1.75 ^H^	0.387 ^H^	0.741	0.862
-Flavanonols	12	4	4	2	2	0.10	0.141	0.68	0.432 ^H^	0.677	0.848
-Flavones	120	67	42	7	4	0.74 ^H^	0.263 ^H^	1.39 ^H^	0.363 ^H^	0.574	0.816
-Flavonols	137	76	47	13	1	0.99 ^H^	0.193 ^H^	1.45 ^H^	0.421 ^H^	0.621	0.840
-Isoflavones	15	12	2	1	0	0.87 ^H^	0.433 ^H^	1.80 ^H^	0.387 ^H^	0.603	0.790
Lignans and Coumarins	101	43	23	27	8	0.30 ^H^	0.370 ^H^	0.54 ^H^	0.372 ^H^	0.697	0.782
Lipids	243	53	40	145	5	0.00	0.221 ^H^	0.47 ^H^	0.346 ^H^	0.531	0.649
Nucleotides and derivatives	78	13	17	43	5	0.54 ^H^	0.382 ^H^	0.05	0.391 ^H^	0.562	0.686
Organic acids	104	11	32	58	3	0.34 ^H^	0.399 ^H^	−0.32	0.366 ^H^	0.683	0.740
Phenolic acids	292	130	91	40	31	0.47 ^H^	0.287 ^H^	0.57 ^H^	0.333 ^H^	0.783	0.804
Quinones	9	5	3	1	0	0.08	0.283	0.87	0.290 ^H^	0.485	0.623
Tannins	4	1	1	2	0	0.64 ^H^	0.268	0.69	0.476 ^H^	0.909	0.881
Terpenoids	133	47	19	17	50	−0.23 ^L^	0.454 ^H^	0.37 ^H^	0.370 ^H^	0.688	0.706
Others	225	78	59	68	20	0.40	0.441 ^H^	−0.18	0.354 ^H^	0.662	0.731
-Alcohol compounds	7	3	2	2	0	0.02	0.333	1.21	0.392 ^H^	0.502	0.498
-Aldehyde compounds	15	6	1	5	3	0.13	0.324 ^H^	−0.50	0.344 ^H^	0.639	0.781
-Chromone	13	5	4	4	0	0.55	0.519 ^H^	0.79	0.343 ^H^	0.614	0.753
-Ketone compounds	21	13	5	2	1	0.62	0.306 ^H^	1.32 ^H^	0.399 ^H^	0.764	0.776
-Lactones	5	1	3	1	0	0.16	−0.191	0.45	0.111	0.622	0.624
-Saccharides	81	11	28	31	11	0.38 ^H^	0.590 ^H^	−1.23 ^L^	0.358 ^H^	0.646	0.707
-Vitamin	22	8	5	9	0	0.91 ^H^	0.289 ^H^	−0.13	0.350 ^H^	0.591	0.740
-Others	61	31	11	14	5	0.28 ^H^	0.420 ^H^	0.32	0.355 ^H^	0.712	0.764
TOTAL	1905	642	466	659	138	0.39 ^H^	0.325 ^H^	0.37 ^H^	0.364 ^H^	0.648	0.743

^1^ Distribution of metabolites into four clusters determined by hierarchical clustering of spatio-temporal data. ^2^ Difference and correlation between metabolites in tip and base of a leaf, and younger (M1 stage) and older (M4 stage) plants. Positive values for difference show higher content of compounds in leaf tip and younger plants, respectively; negative values indicate higher content of compounds in leaf base and older plants, respectively. Data were transformed using inverse normal transformation (INT) before statistical analyses. Letters ‘H’ and ‘L’ indicate the class or subclass of compounds with values significantly (*p* < 0.05) higher (H) or lower (L) than zero. Significance was determined using 10,000 bootstraps. ^3^ Mean broad sense heritability (*H*^2^) of compounds in each class and subclass of metabolites.

## Data Availability

The original contributions presented in this study are included in the article/Supplementary Materials; further inquiries can be directed to the author.

## Supplementary Materials

The following supporting information can be downloaded at: https://www.mdpi.com/article/10.3390/plants13233316/s1, Table S1: Mean values for 1905 metabolites detected in lettuce in experiment 1 (spatio-temporal dynamics) and experiment 2 (accession diversity and metabolites heritability).

## Glossary

Proximodistal—relating to the gradient or differences observed from the base (proximal end) to the tip (distal end) of a leaf.
